# Supplementary figures and images for: Serum IgG titer findings for Fusobacterium nucleatum associated with clinical outcome following surgery in patients with esophageal squamous cell carcinoma
Source: PLoS One. 2025 Nov 21;20(11):e0336219. doi: 10.1371/journal.pone.0336219 (PMC12637919; doi:10.1371/journal.pone.0336219)

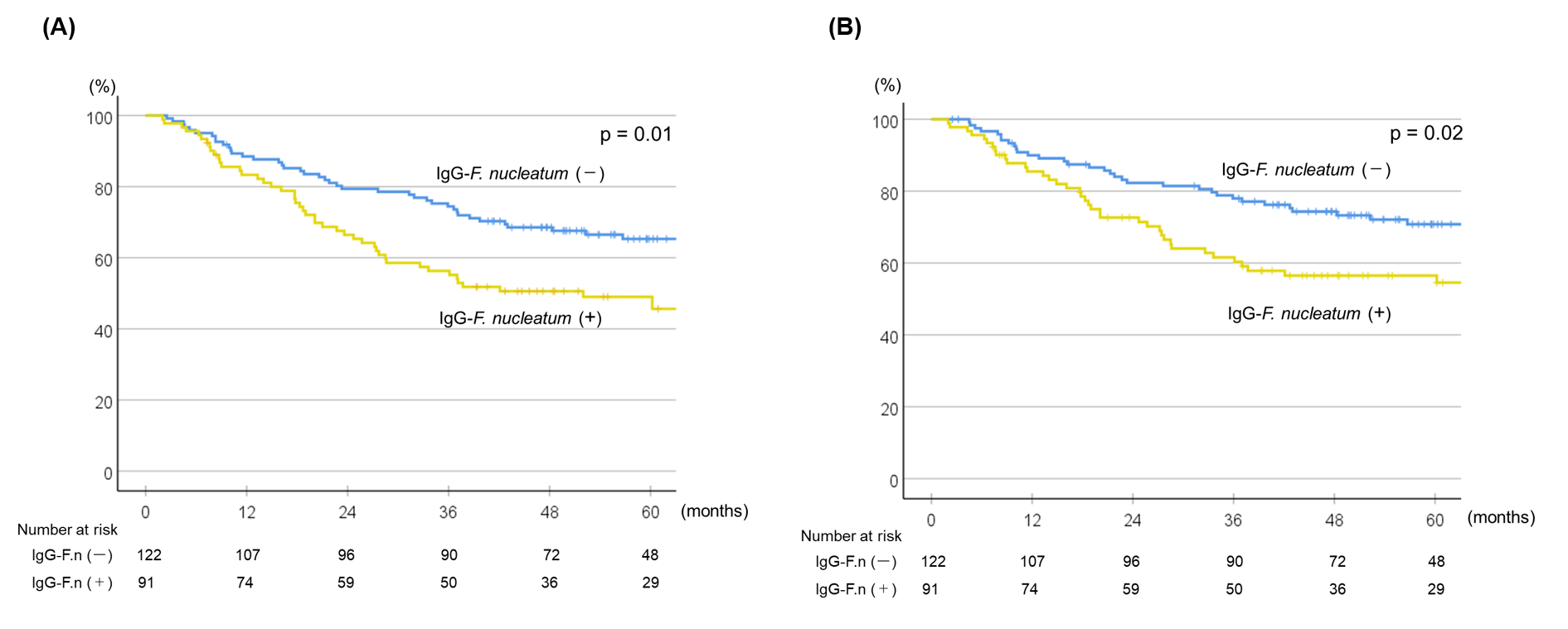

Supplement: S1 Fig — (A) Overall survival (HR 1.68, 95% CI 1.13–2.50; p = 0.01). (B) Cancer-specific survival (HR 1.72, 95% CI 1.10–2.72; p = 0.02). (TIF) [file pone.0336219.s001.tif]

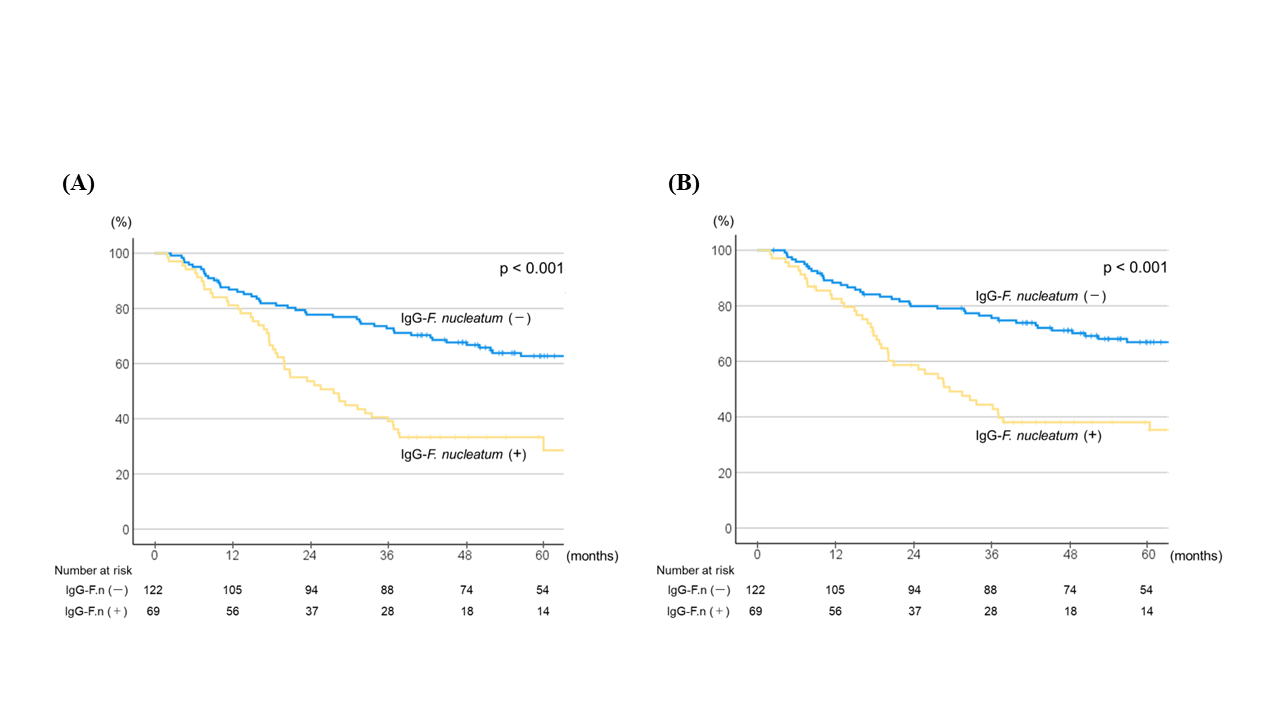

Supplement: S2 Fig — (A) Overall survival (HR 2.28, 95% CI:1.54–3.38; p < 0.001). (B) Cancer-specific survival (HR 2.33, 95% CI 1.52–3.57; p < 0.001). (TIF) [file pone.0336219.s002.tif]
