# Supplementary material for: Serum IgG titer findings for Fusobacterium nucleatum associated with clinical outcome following surgery in patients with esophageal squamous cell carcinoma
Source: PLoS One. 2025 Nov 21;20(11):e0336219. doi: 10.1371/journal.pone.0336219 (PMC12637919; doi:10.1371/journal.pone.0336219)
Supplement: S1 Table — Univariate and multivariate Cox proportional hazards models for cancer-specific survival. Covariates include pre-treatment clinical factors and serum IgG-Fn status. HR > 1 indicates increased hazard of cancer-specific death. (DOCX) [file pone.0336219.s003.docx]

**S1 Table. Cox regression analysis of pretherapeutic factors for cancer-specific survival in patients who underwent oral environment assessment (n = 213).**

| Variables | Univariate analysis | | | Multivariate analysis | | |
| --- | --- | --- | --- | --- | --- | --- |
|  | HR | 95% CI | p | HR | 95% CI | p |
| Age (continuous) | 1.02 | 0.99-1.05 | 0.17 | - | - | - |
| Female (reference: male) | 0.40 | 0.19-0.82 | 0.01* | 0.39 | 0.19-0.83 | 0.01* |
| ECOG PS 1/2  (reference: 0) | 1.96 | 1.23-3.12 | 0.004* | 1.40 | 0.84-2.33 | 0.20 |
| Body mass index (continuous) | 1.00 | 0.93-1.07 | 0.99 | - | - | - |
| Smoking history present (reference: absent) | 1.58 | 0.76-3.29 | 0.22* | - | - | - |
| Alcohol consumption present (reference: absent) | 1.88 | 0.76-4.65 | 0.17* | - | - | - |
| Diabetes Mellitus present (reference: absent) | 1.17 | 0.62-2.21 | 0.64 | - | - | - |
| Upper tumor location (reference: middle, lower, or EGJ) | 0.99 | 0.53-1.83 | 0.97 | - | - | - |
| Poor differentiation shown by biopsy  (reference: others) | 1.87 | 1.11-3.14 | 0.02* | 1.41 | 0.82-2.43 | 0.22 |
| CEA^a^ >5 (reference: normal value ≤5) | 1.04 | 0.55-1.98 | 0.90 | - | - | - |
| SCC^a^ >1.5 (reference: normal value ≤1.5) | 1.06 | 0.66-1.69 | 0.81 | - | - | - |
| Neoadjuvant therapy present (reference: absent) | 2.37 | 1.30-4.31 | 0.01* | 1.11 | 0.53-2.30 | 0.79 |
| cT^b^ 3/4 (reference: 1/2) | 4.62 | 2.43-8.78 | <0.001* | 3.53 | 1.34-9.27 | 0.01* |
| cN^b^ 1/2/3 (reference: 0) | 3.28 | 1.89-5.71 | <0.001* | 1.73 | 0.44-6.89 | 0.44 |
| cM (LYM)^b, c^ 1 (reference: 0) | 2.14 | 1.15-3.97 | 0.02* | 1.32 | 0.69-2.55 | 0.40 |
| cStage^b^ III/IV (reference: I/II) | 3.57 | 2.12-6.01 | <0.001* | 0.89 | 0.21-3.81 | 0.87 |
| Lost tooth number ≥8 (reference: <8) | 1.17 | 0.74-1.86 | 0.50 | - | - | - |
| Bleeding on probing (%) ≥30 (reference: <0) | 0.91 | 0.58-1.43 | 0.68 | - | - | - |
| IgG-*Fn* positive (reference: negative) | 1.81 | 1.15-2.86 | 0.01* | 2.22 | 1.40-3.53 | <0.001* |

Univariate and multivariate Cox proportional hazards models for cancer-specific survival. Covariates include pre-treatment clinical factors and serum IgG-Fn status. HR>1 indicates increased hazard of cancer-specific death. CEA, carcinoembryonic antigen; CI, confidence interval; ECOG PS, Eastern Cooperative Oncology Group performance status; EGJ, esophagogastric junction; Fn, Fusobacterium nucleatum; HR, hazard ratio; IgG, immunoglobulin G; SCC, squamous cell carcinoma-related antigen.

a　Pre-treatment data.

b　Pre-therapeutic staging according to TNM Classification, 8th edition.

c　cM1(LYM), clinical metastasis to supraclavicular lymph node.

* p< 0.05 indicates significance.
