# Supplementary material for: Serum IgG titer findings for Fusobacterium nucleatum associated with clinical outcome following surgery in patients with esophageal squamous cell carcinoma
Source: PLoS One. 2025 Nov 21;20(11):e0336219. doi: 10.1371/journal.pone.0336219 (PMC12637919; doi:10.1371/journal.pone.0336219)
Supplement: S2 Table — Clinicopathological characteristics of ESCC patients receiving neoadjuvant therapy based on IgG-Fn titer status, with TNM staging based on the TNM Classification, 8th edition. ypStage 0 and ypT0 denotes complete pathological response, with ypStage 0 showing no lymph node metastasis. pM1(LYM) denotes metastasis to the supraclavicular lymph node. p < 0.05 indicates significance. (DOCX) [file pone.0336219.s004.docx]

**S2 Table. Clinicopathological characteristics of ESCC patients undergoing neoadjuvant therapy by IgG-Fn titer (n = 191).**

| Clinical parameters | Serum IgG titer for *Fn* | | *p* |
| --- | --- | --- | --- |
|  | Negative  n = 122 | Positive  n = 69 |  |
| Age, mean ± SD | 65.0 ± 8.2 | 66.8 ± 7.6 | 0.15 |
| Sex, (%) |  |  |  |
| Male | 97 (79.5) | 59 (85.5) | 0.34 |
| Female | 25 (20.5) | 10 (14.5) |  |
| ECOG PS, (%) |  |  |  |
| 0 | 88 (72.1) | 46 (66.7) | 0.51 |
| 1, 2 | 34 (27.9) | 23 (33.3) |  |
| Smoking history, (%) |  |  |  |
| Yes | 103 (84.4) | 63 (91.3) | 0.26 |
| No | 19 (15.6) | 6 (8.7) |  |
| History of alcohol consumption, (%) |  |  |  |
| Yes | 105 (84.4) | 63 (84.4) | 0.36 |
| No | 17 (84.4) | 6 (84.4) |  |
| Diabetes mellitus, (%) |  |  |  |
| Present | 12 (9.8) | 11 (15.9) | 0.25 |
| Absent | 110 (90.2) | 58 (84.1) |  |
| Tumor marker (pre-treatment), mean ± SD |  |  |  |
| SCC, ng/mL | 2.1 ± 2.8 | 2.7 ± 3.3 | 0.13 |
| CEA, ng/mL | 3.0 ± 1.9 | 3.5 ± 3.1 | 0.15 |
| Tumor location, (%) |  |  |  |
| Upper third | 24 (19.7) | 8 (11.6) | 0.23 |
| Middle third | 57 (46.7) | 40 (58.0) |  |
| Lower third/EGJ | 41 (33.6) | 21 (30.4) |  |
| Tumor differentiation by biopsy, (%) |  |  |  |
| Poor | 30 (24.6) | 14 (20.3) | 0.59 |
| Other | 92 (75.4) | 55 (79.7) |  |
| Tumor depth, (%) |  |  |  |
| cT1/2 | 28 (22.9) | 11 (16.0) | 0.27 |
| cT3/4 | 94 (77.1) | 58 (84.0) |  |
| Lymph node metastasis, (%) |  |  |  |
| cN0 | 32 (26.2) | 16 (23.2) | 0.73 |
| cN1-3 | 90 (73.8) | 53 (76.8) |  |
| Distant metastasis, (%) (cM1: supraclavicular) |  |  |  |
| cM0 | 109 (89.3) | 61 (88.4) | 0.82 |
| cM1 (LYM) | 13 (10.7) | 8 (11.6) |  |
| TNM stage, (%) |  |  |  |
| cStage II | 40 (32.8) | 18 (26.1) | 0.41 |
| cStage III/IV | 82 (67.2) | 51 (73.9) |  |
| Neoadjuvant therapy, (%) |  |  |  |
| Chemotherapy | 49 (40.2) | 34 (49.3) | 0.23 |
| Chemoradiotherapy | 73 (59.8) | 35 (50.7) |  |
| Oral environment (pre-treatment), mean ± SD | (n = 91) | (n = 61) |  |
| Tooth loss | 8.7 ± 9.4 | 6.8 ± 7.4 | 0.16 |
| Bleeding on probing | 35.3 ± 32.4 | 32.4 ± 24.3 | 0.54 |
| Pathological parameters | | | |
| Tumor depth, (%) |  |  |  |
| ypT0-2 | 77 (63.1) | 33 (47.8) | <0.05* |
| ypT3/4 | 45 (36.9) | 36 (52.2) |  |
| Lymph node metastasis, (%) |  |  |  |
| ypN0 | 64 (52.5) | 23 (33.3) | 0.02* |
| ypN1-3 | 58 (47.5) | 46 (66.7) |  |
| Distant metastasis, (%) |  |  |  |
| ypM0 | 116 (95.1) | 62 (89.9) | 0.23 |
| ypM1(LYM) | 6 (4.9) | 7 (10.1) |  |
| TNM stage, (%) |  |  |  |
| ypStage 0-II | 77 (63.1) | 25 (36.2) | < 0.001* |
| ypStage III/IV | 45 (36.9) | 44 (63.8) |  |
| Pathological regression grade of primary tumor, (%) | |  |  |
| 0 | 1 (0.8) | 5 (7.2) | 0.01* |
| 1 | 41 (33.6) | 31 (44.9) |  |
| 2 | 44 (36.1) | 23 (33.3) |  |
| 3 | 36 (29.5) | 10 (14.5) |  |

Clinicopathological characteristics of ESCC patients receiving neoadjuvant therapy based on IgG-Fn titer status, with TNM staging based on the TNM Classification, 8th edition. ypStage 0 and ypT0 denotes complete pathological response, with ypStage 0 showing no lymph node metastasis. pM1(LYM) denotes metastasis to the supraclavicular lymph node. * p< 0.05 indicates significance.
