# Supplementary material for: Serum IgG titer findings for Fusobacterium nucleatum associated with clinical outcome following surgery in patients with esophageal squamous cell carcinoma
Source: PLoS One. 2025 Nov 21;20(11):e0336219. doi: 10.1371/journal.pone.0336219 (PMC12637919; doi:10.1371/journal.pone.0336219)
Supplement: S3 Table — Logistic regression analyses of pretherapeutic factors for pathological primary tumor response (Grade 2/3), based on the response evaluation criteria of the Japan Esophageal Society. p < 0.05 indicates statistical significance. CI, confidence interval; OR, Odds ratio. (DOCX) [file pone.0336219.s005.docx]

**S3 Table. Logistic regression analysis of pre-therapeutic factors for pathological tumor response to neoadjuvant therapy (TRG 2–3 vs 0–1) in stage II–IV ESCC patients (n = 191).**

| Variables | Univariate analysis | | | Multivariate analysis | | |
| --- | --- | --- | --- | --- | --- | --- |
|  | OR | 95% CI | *p* | OR | 95% CI | *p* |
| Age (continuous) | 1.01 | 0.98-1.05 | 0.47 | - | - | - |
| Female (reference: male) | 1.93 | 0.87-4.29 | 0.11 | - | - | - |
| ECOG PS 1/2 (reference: 0) | 0.93 | 0.50-1.74 | 0.82 | - | - | - |
| Body mass index (continuous) | 1.02 | 0.93-1.12 | 0.70 | - | - | - |
| Smoking history present (reference: absent) | 0.65 | 0.26-1.57 | 0.34 | - | - | - |
| Alcohol consumption present (reference: absent) | 0.60 | 0.23-1.53 | 0.28 | - | - | - |
| Diabetes mellitus present (reference: absent) | 1.08 | 0.44-2.65 | 0.86 | - | - | - |
| Upper tumor location (reference: middle, lower or EGJ) | 0.61 | 0.27-1.37 | 0.23 | - | - | - |
| Poor differentiation shown in biopsy findings (reference: others) | 0.78 | 0.39-1.57 | 0.49 | - | - | - |
| CEA (pre-treatment)> 5 (reference: normal value ≤ 5) | 1.36 | 0.57-3.23 | 0.49 | - | - | - |
| SCC (pre-treatment)> 1.5 (reference: normal value ≤ 1.5) | 0.78 | 0.44-1.41 | 0.41 | - | - | - |
| cT 3/4 (reference: 1/2) | 0.88 | 0.43-1.82 | 0.74 | - | - | - |
| cN 1/2/3 (reference: 0) | 1.05 | 0.54-2.03 | 0.89 | - | - | - |
| cM (LYM) 1 (reference: 0) | 0.91 | 0.36-2.28 | 0.84 | - | - | - |
| cStage III/IV (reference: I/II) | 0.93 | 0.50-1.75 | 0.83 | - | - | - |
| Neoadjuvant chemoradiotherapy (reference: chemotherapy) | 9.08 | 4.67-17.66 | <0.001* | 9.06 | 4.62-17.80 | <0.001* |
| Tooth loss (Pre-treatment) ≥8 (reference: <8) | 0.81 | 0.42-1.57 | 0.54 | - | - | - |
| Bleeding on probing (pre-treatment) (%) ≥30 (reference: < 0) | 0.52 | 0.26-1.01 | 0.07 | - | - | - |
| IgG-*Fn* positive (reference: negative) | 0.48 | 0.26-0.88 | 0.02* | 0.48 | 0.24-0.97 | 0.04* |

Logistic regression analyses of pretherapeutic factors for pathological primary tumor response (Grade 2/3), based on the response evaluation criteria of the Japan Esophageal Society. CI, confidence interval; OR, Odds ratio.

* p< 0.05 indicates significance.
