# Supplementary material for: Serum IgG titer findings for Fusobacterium nucleatum associated with clinical outcome following surgery in patients with esophageal squamous cell carcinoma
Source: PLoS One. 2025 Nov 21;20(11):e0336219. doi: 10.1371/journal.pone.0336219 (PMC12637919; doi:10.1371/journal.pone.0336219)
Supplement: Attachment — Submitted filename: Response to Reviewers .docx [file pone.0336219.s006.docx]

Professor David M. Ojcius

Academic Editor

*PLOS ONE*

Re: “**Serum IgG titer findings for *Fusobacterium nucleatum* associated with clinical outcome following surgery in patients with esophageal squamous cell carcinoma**”

Dear Professor Ojcius,

Thank you very much for your email dated September 18, 2025, along with the excellent reviewer comments and suggestions regarding our manuscript. We have incorporated these in the revised version, which you will find attached. Thank you for providing us with an opportunity to resubmit our study and we trust that it will be found acceptable for publication in *PLOS ONE.* Following are our detailed replies.

・・・・・・・・・・・・・・・・・・・・・・・・・・・・・・・・・・・・・・・・・・・・・・・・・・・・・・・・・・・・・・

**Comment 1**

**The ELISA methodology requires significant elaboration to ensure reproducibility. Specifically, authors should detail the rationale for selecting the F. nucleatum subspecies beyond citing ATCC 10953, explain how serum dilution ratios were optimized. Clarity regarding sample timing is essential—explicitly confirm all specimens were obtained before any treatment initiation, including neoadjuvant therapy. The partial oral health assessment in just 213 patients introduces possible selection bias that warrants explanation or sensitivity analysis.**

**Authors’ response:**

Thank you for these valuable suggestions. The rationale for selecting *ATCC 10953 F. nucleatum subsp. polymorphum)* among the four subspecies of *Fusobacterium nucleatum* for ELISA and steps taken to adopt a 1:3200 dilution is now described in the revised Methods section (lines 366-369, 373-375). In addition, to clarify the timing of blood collection, it is noted that it was performed before the start of any treatment in all cases (Methods, lines 361-362).

Based on these excellent comments and suggestions, results of sensitivity analysis regarding the response to partial oral health assessment have been added to the revised manuscript. Similar to the main analysis, the findings obtained with the subset of 213 patients showed that IgG-*Fn* positivity was consistently associated with poor prognosis (OS: HR=1.68, 95% CI 1.13-2.50; p=0.01) (CSS: HR=1.72, CI 1.10-2.72; p=0.02) (multivariate Cox CSS: HR=2.22, CI 1.40-3.53; p<0.001). These results have been added to the Methods (lines 417-418) and Results (lines 139-152) sections and are also presented in Supplementary Figure 1 and Supplementary Table 1.

**Comment 2**

**Statistical reporting needs refinement throughout. All Kaplan-Meier figures must be supplemented with "Number at Risk" tables and annotated with hazard ratios to improve interpretability. Critical inconsistencies between text sections require resolution, such as the abstract's misstatement about IgG-Fn-negative patients showing worse pathology—this directly contradicts Supplementary Table 1 findings where IgG-Fn-positive status correlates with adverse pathological features.**

**Authors’ response:**

We appreciate these helpful comments. We have added number at risk indicating results of Kaplan-Meier analyses to all the figures and also added hazard ratios to the figure legends (lines 102-133). In addition, the misstatement in the original Abstract has been corrected (line 34).

**Comment 3**

**Interpretation would benefit from deeper biological contextualization. The stark contrast in IgG-Fn's prognostic significance between Stage I versus advanced-stage patients necessitates mechanistic exploration—is this attributable to statistical power limitations, fundamental biological differences in early disease, or the modifying effect of neoadjuvant therapy exposure? While chemotherapy resistance mechanisms are cited, the discussion fails to integrate how the IgG biomarker itself relates to these pathways—does elevated IgG indicate failed bacterial clearance that enables Fn-mediated treatment resistance?**

**Authors’ response:**

Thank you for these insightful comments. The following has been added to the Discussion section in the revised version.

Notably, the prognostic impact of IgG-*Fn* was found to vary according to stage. In Stage I patients, the survival rate was high and number of events was low, thus limiting the ability to detect a significant effect. In contrast, in advanced clinical stages, tumor-associated inflammation and immunosuppression were more pronounced. Thus, sustained *Fn* exposure in such cases may promote immune dysregulation and chemotherapy resistance, leading to enhancement of the prognostic significance of IgG-Fn. (lines 292-298).

An elevated anti-*Fn* IgG level suggests prolonged exposure to *Fn* or persistence in the host rather than a simple past infection. This may activate cell survival-related pathways such as inflammatory signaling and autophagy, possibly resulting in reduced sensitivity to cytotoxic therapy. (lines 251-254).
